# Association of Alcohol Drinking Patterns With Presence of Impaired Fasting Glucose and Diabetes Mellitus Among South Korean Adults

**DOI:** 10.2188/jea.JE20170021

**Published:** 2018-03-05

**Authors:** Jisun Lim, Jung Ah Lee, Hong-Jun Cho

**Affiliations:** Department of Family Medicine, Asan Medical Center, University of Ulsan College of Medicine, Seoul, South Korea

**Keywords:** alcohol drinking pattern, impaired fasting glucose, diabetes mellitus

## Abstract

**Background:**

We aimed to investigate the association between alcohol drinking patterns and the presence of impaired fasting glucose (IFG) and diabetes mellitus (DM).

**Methods:**

We used data from the Korean National Health and Nutrition Examination Survey, 2010–2014. The participants were aged ≥30 years and had no previous diagnosis of DM. High-risk drinking was defined as alcohol consumption of ≥7 glasses at a sitting for men, and ≥5 glasses for women. After adjusting for confounding factors, a polychotomous logistic regression analysis was performed to assess the association of drinking patterns with IFG and DM.

**Results:**

For men, high-risk drinking was associated with higher odds ratios (ORs) of IFG (2–4/month, OR 1.51; 95% confidence interval [CI], 1.13–2.04; 2–3/week, OR 1.79; 95% CI, 1.38–2.33; and ≥4/week, OR 2.24; 95% CI, 1.65–3.03) and of DM (2–4/month, OR 2.12; 95% CI, 1.20–3.77; 2–3/week, OR 1.78; 95% CI, 1.05–3.03; and ≥4/week, OR 2.98; 95% CI, 1.72–5.17). For women, high-risk drinking was associated with higher risk of IFG (2–4/month, OR 1.51; 95% CI, 1.04–2.21; 2–3/week, OR 3.19; 95% CI, 2.20–4.64; and ≥4/week, OR 2.23; 95% CI, 1.23–4.06), but not of DM, compared with non-high-risk drinkers who consumed alcohol ≤1 day/month. Non-high-risk drinkers who consumed alcohol ≥4 days/week had higher ORs of DM in men, but lower ORs of DM in women compared with non-high risk drinkers who consumed alcohol ≤1 day/month.

**Conclusions:**

Compared with non-high-risk alcohol drinking, even occasional high-risk alcohol drinking was associated with a higher risk of IFG in men and women, and DM in men. Nearly daily non-high-risk alcohol drinking was associated with a higher risk of DM in men and lower risk of DM in women.

## INTRODUCTION

Diabetes mellitus (DM) is a disease with increasing incidence worldwide. The International Diabetes Federation announced in 2015 that 1 in 11 adults (415 million) worldwide have diabetes. According to a report of the Korea Health Statistics (2014) with nationally representative data from the Korean National Health and Nutrition Examination Survey (KNHANES), the prevalence of DM in Korean adults aged ≥30 years was approximately 9.0% in 2005 and had increased to 10.2% in 2014.

DM is a risk factor for cardiovascular and cerebrovascular diseases.^1^ Mortality and morbidity from cardiovascular disease in patients with DM are 2- to 3-fold higher in men and 3- to 5-fold higher in women than in persons without DM.^2^^,^^3^ The microvascular and macrovascular complications of diabetes also make DM a leading cause of death in patients with this disease, negatively affecting both health and life expectancy.^4^

Although a family history of diabetes is an established risk factor for type 2 DM, some lifestyle behaviors, such as low levels of physical activity and consumption of high-calorie or high-fat diets leading to obesity, have been shown to increase the risk of type 2 DM.^5^ An association between average alcohol consumption and risk of type 2 DM has been demonstrated, with many studies reporting a J- or U-shaped association. Carlsson et al^6^ found that moderate alcohol consumption was associated with a reduced risk of type 2 DM in both men and women, whereas high alcohol consumption might increase the risk of DM in women. Another prospective study of 8,663 men reported a U-shaped association between alcohol intake and diabetes, with an elevated risk of developing type 2 diabetes in nondrinkers and men with high alcohol intakes when compared with men who reported moderate alcohol intake.^7^

The pattern of alcohol consumption may also contribute to the development of diabetes. However, findings from several studies on the association between alcohol drinking patterns and diabetes have been mixed. In one study, the risk of diabetes increased significantly in men who drank heavily (>3 drinks in a sitting) compared with men with <1 drink per sitting, regardless of drinking frequency.^8^ However, in another study, men who consumed approximately 48 g ethanol 4–7 times per week had a decreased risk of type 2 diabetes when compared with nondrinkers, although the difference was not significant.^9^ One prospective study also showed that frequent alcohol consumption was associated with a decreased risk of diabetes, even in men who consumed >3 drinks per day.^10^ In using the Alcohol Use Disorders Identification Test (AUDIT), one Korean study showed that a high AUDIT score (≥15) was associated with an increased risk of DM in men but not in women.^11^ However, this study did not take into account impaired fasting glucose (IFG), which represents a prediabetic state.^12^ Moreover, since the AUDIT score includes an assessment of dependency symptoms in addition to alcohol consumption, the frequency of high-risk drinking may be a more accurate indicator of the degree of binge drinking than the AUDIT score.

This study aimed to investigate the association of alcohol drinking patterns, such as frequency of high-risk alcohol consumption, with the presence of IFG and DM using nationally representative data.

## METHODS

### Study subjects

This study was based on data collected in KNHANES 2010–2014. KNHANES is a cross-sectional, nationally representative survey conducted by the Division of Chronic Disease Surveillance, Korea Centers for Disease Control and Prevention. Household units were selected by using stratified, multistage, clustered probability sampling according to geographic area, sex, and age group.^13^

From 2010 to 2012 (V) and 2013 to 2014 (VI), 192 primary sampling units were proportionally allocated to reflect the target population, and 3,840 households were investigated each year. The selected samples were weighted to represent the entire Korean population. The surveys consisted of a health interview survey, a nutrition survey, and a health examination survey. Data were collected through household interviews, and direct standardized physical examinations were performed at specially equipped mobile examination centers. From the total KNHANES sample (*n* = 41,102), we used the data for persons aged ≥30 years (*n* = 27,887) and excluded those in whom DM had been diagnosed by doctors (*n* = 2,420) in order to avoid the sick quitter effect. Subjects whose records were incomplete (*n* = 6,619) were also excluded. Our final study sample included 18,848 participants (7,875 men and 10,973 women). All survey participants provided informed consent. Ethical approval was not required because the study was a retrospective analysis of a national surveillance data set that did not contain personally identifiable information.

### Measurements and definitions of variables

Household income was grouped into low, low-middle, middle-high, and high quartiles. Education level was classified as ≤6, 7–9, 10–12, and ≥13 years. Participants were considered to have low levels of aerobic physical activity if they did not engage in high-intensity activities for >75 min/week or moderate-intensity activities for >150 min/week. High-intensity activities included jogging, fast swimming, skipping, or squash. Moderate-intensity activities included tennis, slow swimming, badminton, or table tennis. According to smoking status, the participants were classified as “current smokers,” “ex-smokers,” or “never smokers.” The participants were considered to have a family history of diabetes if DM had been diagnosed by doctors in either their father or mother.

Body mass index (BMI) was calculated by dividing body weight (kg) by the square of height (m^2^). Body weight was measured to the nearest 0.1 kg on a balanced scale, and height was measured to the nearest 0.1 cm while the participants wore a light gown without shoes. Participants with BMI <18.5 kg/m^2^ were considered underweight, those with BMI 18.5–24.9 kg/m^2^ were considered to have a normal body mass, and those with BMI ≥25.0 kg/m^2^ were considered overweight according to the World Health Organization (WHO) definition of obesity.^14^

Blood samples were collected from the antecubital vein in the morning after an overnight fast. The concentrations of serum glucose and total cholesterol were measured with a Hitachi Automatic Analyzer 7600 (Tokyo, Japan) and hemoglobin A1c (HbA1c) was quantified using HLC-723G7 (Tosoh, Tokyo, Japan). Participants were considered hypercholesterolemic if their total cholesterol level was >240 mg/dL (measured after an 8-hr fast) or if they were taking cholesterol-lowering medications. DM was defined as serum glucose ≥126 mg/dL after an 8-hr fast or HbA1c ≥6.5%, while IFG was defined as fasting serum glucose between 100 and 126 mg/dL. Participants with fasting serum glucose <100 mg/dL were considered to have normal serum glucose.

Alcohol consumption was assessed through questioning of the participants about their drinking behavior in the last 12 months, and was categorized into drinking frequency (never, less than once a month, once a month, 2–4 times a month, 2–3 times a week, or ≥4 times a week), and quantity consumed per each drinking day (1–2, 3–4, 5–6, 7–9, or ≥10 glasses). The amount in a glass was calculated without distinction between *soju* (Korean distilled spirit) and foreign spirits. One can (355 mL) of beer was considered equal to 1.6 glasses of beer. About 10 g alcohol was included in one glass. The average alcohol consumption (number of glasses) in 1 month was calculated by multiplying the drinking frequency in 1 month by the quantity consumed per each drinking day, and using the midpoints of the categories. Average alcohol consumption (in g/month) was calculated by multiplying the average alcohol consumption (number of glasses) in 1 month by 10 g. For open-ended top categories (eg, ≥10 glasses), we followed other analysts by adding three quarters of the range of the previous category to the lower bound.^15^ High-risk drinking was defined according to the WHO definition of daily alcohol consumption (≥60 g for men and ≥40 g for women) as alcohol consumption of ≥7 glasses (or 5 cans of beer) on a single occasion for men and ≥5 glasses for women.^16^

### Statistical analysis

Descriptive statistics and general linear models were used to analyze continuous variables, and data are presented as means (standard errors). The chi-square test was used to analyze categorical variables, and data are presented as unweighted numbers (weighted percentages). The odds ratios (ORs) and 95% confidence intervals (CIs) for IFG and DM were analyzed according to average alcohol consumption and drinking patterns using polychotomous logistic regression analysis. Model 1 was adjusted for age, and model 2 was adjusted for age, BMI, smoking status, household income, educational level, physical activity, family history of DM, and hypercholesterolemia. Additionally, the quantity consumed per each drinking day was adjusted to analyze the ORs of IFG and DM according to drinking patterns. All analyses were conducted using SPSS Statistics version 21.0 (SPSS Inc., Chicago, IL, USA), and weighted to represent the Korean population. A two-sided *P* value of 0.05 was considered statistically significant.

## RESULTS

Table 1 shows the patterns of alcohol consumption and the characteristics of the study subjects. In men, the mean age of lifetime abstainers (54.8 years), past drinkers (53.6 years), and frequent (≥4/week) non-high-risk drinkers (55.7 years) were significantly higher than that of other drinkers. The percentages of lowest household income, lowest educational status, no physical activity, and underweight were highest in lifetime abstainers. Among drinkers, the percentages of DM, lowest household income, and lowest educational status were highest in the most frequent non-high-risk drinkers, and the percentages of IFG and current smoking were highest in the most frequent high-risk drinkers. In women, the mean age of lifetime abstainers (58.1 years) was highest. The percentages of DM, lowest household income, and lowest educational status were highest in lifetime abstainers. Among drinkers, the percentage of DM was highest in groups with a frequency of non-high-risk drinking of ≤1 per month and 2–3/week, and the percentage of IFG was highest in groups with a frequency of high-risk drinking of 2–3 per week. The percentages of lowest household income, lowest educational status, and hypercholesterolemia were highest in the most frequent non-high-risk drinkers, and the percentage of current smoking was highest in the most frequent high-risk drinkers.

**Table 1.** Patterns of alcohol consumption and characteristics of the study subjects

Men											

Alcohol consumption:			Frequency of non-high-risk drinking				Frequency of high-risk drinking^a^				*P* value^b^
	
	Lifetime abstainer	Past drinker	≤1/month	2–4/month	2–3/week	≥4/week	≤1/month	2–4/month	2–3/week	≥4/week	
	(*n* = 386)	(*n* = 868)	(*n* = 1,255)	(*n* = 1,285)	(*n* = 995)	(*n* = 552)	(*n* = 165)	(*n* = 775)	(*n* = 1,031)	(*n* = 563)	
Age, years	54.8 (0.9)	53.6 (0.5)	47.3 (0.4)	48.0 (0.4)	50.7 (0.4)	55.7 (0.7)	42.3 (0.8)	41.5 (0.4)	43.2 (0.3)	48.3 (0.5)	<0.001
Average alcohol consumption, g/month	—	—	19.5 (0.5)	118.2 (1.5)	427.5 (5.3)	803.8 (14.9)	87.5 (3.7)	352.1 (5.0)	1,231.9 (14.7)	2,492.4 (37.4)	<0.001
DM status											<0.001
DM	21 (6.6)	55 (6.2)	56 (3.9)	70 (5.8)	52 (5.0)	47 (10.8)	6 (2.6)	41 (5.4)	49 (5.1)	53 (8.9)	
IFG	86 (20.6)	236 (25.9)	299 (23.2)	327 (24.1)	318 (29.9)	180 (29.9)	37 (18.9)	204 (25.4)	319 (30.9)	217 (36.5)	
Normal	279 (72.8)	577 (67.8)	900 (72.9)	888 (70.1)	625 (65.1)	325 (59.3)	122 (78.5)	530 (69.2)	663 (64.0)	293 (54.6)	
Household income											<0.001
Low	113 (22.5)	235 (20.9)	165 (10.5)	159 (9.6)	153 (12.1)	146 (19.6)	16 (8.1)	39 (5.0)	63 (5.7)	89 (12.7)	
Low-middle	105 (29.8)	239 (28.6)	343 (26.1)	303 (23.3)	264 (27.3)	162 (30.5)	42 (23.4)	171 (24.2)	232 (23.0)	156 (28.1)	
Middle-high	98 (27.3)	219 (28.3)	372 (32.0)	388 (31.6)	282 (30.3)	133 (27.1)	71 (48.6)	256 (32.8)	338 (34.7)	153 (27.8)	
High	70 (20.4)	175 (22.2)	375 (31.3)	435 (35.6)	296 (30.4)	111 (22.8)	36 (20.0)	309 (38.1)	398 (36.5)	165 (31.3)	
Education status											<0.001
≤6 years	132 (26.6)	235 (19.7)	159 (9.4)	145 (8.5)	184 (15.8)	177 (23.8)	16 (5.7)	27 (2.9)	54 (4.0)	110 (15.8)	
7–9 years	54 (11.3)	118 (13.5)	142 (9.7)	138 (10.0)	147 (13.3)	108 (19.9)	11 (5.8)	32 (3.8)	82 (8.5)	89 (16.2)	
10–12 years	98 (27.1)	247 (31.5)	424 (37.2)	437 (36.3)	352 (36.6)	161 (34.0)	37 (27.2)	248 (33.3)	398 (41.4)	198 (36.8)	
≥13 years	102 (35.0)	268 (35.3)	530 (43.7)	565 (45.2)	312 (34.3)	106 (22.2)	101 (61.4)	468 (60.1)	497 (46.2)	166 (31.1)	
Physical activity											0.032
Yes	130 (36.5)	326 (43.4)	522 (42.9)	577 (46.6)	436 (45.5)	241 (45.7)	66 (41.4)	366 (46.3)	494 (49.5)	244 (45.0)	
No	256 (63.5)	542 (56.6)	733 (57.1)	708 (53.4)	559 (54.5)	311 (54.3)	99 (58.6)	409 (53.7)	537 (50.5)	319 (55.0)	
Smoking status											<0.001
Current smoker	104 (30.0)	200 (25.1)	447 (41.1)	460 (39.2)	408 (46.0)	218 (44.5)	72 (44.1)	402 (55.3)	606 (61.4)	333 (63.4)	
Ex-smoker	114 (23.4)	479 (50.7)	515 (34.7)	560 (38.5)	431 (38.0)	282 (46.0)	60 (35.5)	279 (33.6)	309 (27.7)	201 (31.4)	
Never smoker	168 (46.6)	189 (24.1)	293 (24.2)	265 (22.2)	156 (16.0)	52 (9.5)	33 (20.3)	94 (11.2)	116 (10.9)	29 (5.2)	
BMI											<0.001
<18.5 kg/m^2^	20 (4.5)	30 (2.9)	38 (3.2)	25 (1.6)	29 (2.8)	12 (1.8)	2 (1.0)	10 (1.3)	12 (1.1)	6 (0.9)	
18.5–24.9 kg/m^2^	242 (59.0)	564 (63.0)	790 (61.4)	841 (65.0)	643 (62.7)	391 (71.1)	90 (52.7)	390 (51.0)	525 (49.7)	315 (55.9)	
≥25 kg/m^2^	124 (36.5)	274 (34.1)	427 (35.4)	419 (33.4)	323 (34.4)	149 (27.1)	73 (46.3)	375 (47.6)	494 (49.2)	242 (43.1)	
Hypercholesterolemia											0.354
Yes	48 (12.3)	118 (13.2)	138 (10.9)	132 (10.0)	138 (13.8)	70 (13.8)	18 (10.7)	90 (11.4)	142 (13.2)	72 (12.7)	
No	338 (87.7)	750 (86.8)	1,117 (89.1)	1,153 (90.0)	857 (86.2)	482 (86.2)	147 (89.3)	685 (88.6)	889 (86.8)	491 (87.3)	
Family history of DM											<0.001
Yes	35 (10.8)	94 (13.1)	196 (17.8)	188 (17.0)	133 (15.2)	48 (9.9)	33 (22.0)	151 (20.6)	203 (20.6)	92 (18.0)	
No	351 (89.2)	774 (86.9)	1,059 (82.2)	1,097 (83.0)	862 (84.8)	504 (90.1)	132 (78.0)	624 (79.4)	828 (79.4)	471 (82.0)	

Women											

			Frequency of non-high risk drinking				Frequency of high risk drinking				*P* value^b^
	
	Lifetime abstainer	Past drinker	≤1/month	2–4/month	2–3/week	≥4/week	≤1/month	2–4/month	2–3/week	≥4/week	
	(*n* = 2,073)	(*n* = 1,928)	(*n* = 3,799)	(*n* = 1,439)	(*n* = 480)	(*n* = 124)	(*n* = 272)	(*n* = 440)	(*n* = 307)	(*n* = 111)	

Age, years	58.1 (0.4)	50.5 (0.4)	48.2 (0.2)	46.3 (0.4)	45.9 (0.6)	52.7 (1.4)	42.4 (0.7)	41.8 (0.5)	41.6 (0.6)	43.5 (1.0)	<0.001
Average alcohol consumption, g/month	—	—	12.5 (0.2)	73.6 (1.0)	262.5 (5.3)	503.5 (21.8)	57.4 (2.2)	228.0 (6.2)	831.7 (28.5)	2,103.8 (105.2)	<0.001
DM status											<0.001
DM	126 (6.3)	87 (4.7)	162 (3.9)	50 (2.9)	18 (3.9)	2 (0.9)	6 (1.7)	13 (2.9)	7 (2.5)	3 (2.3)	
IFG	445 (21.4)	308 (15.8)	608 (15.4)	263 (18.1)	89 (17.6)	26 (18.6)	40 (12.8)	72 (17.3)	83 (29.3)	26 (23.8)	
Normal	1,502 (72.2)	1,533 (79.5)	3,029 (80.7)	1,126 (78.9)	373 (78.5)	96 (80.5)	226 (85.5)	355 (79.8)	217 (68.2)	82 (73.9)	
Household income											<0.001
Low	641 (27.9)	413 (19.3)	524 (12.4)	193 (11.6)	61 (10.6)	34 (25.5)	39 (14.1)	35 (7.2)	37 (12.3)	16 (12.1)	
Low-middle	549 (27.0)	527 (30.0)	988 (26.7)	307 (22.7)	109 (23.4)	33 (27.2)	58 (21.6)	113 (30.1)	106 (35.7)	30 (28.3)	
Middle-high	431 (22.6)	479 (24.9)	1,097 (29.6)	458 (33.2)	160 (34.7)	32 (22.1)	99 (36.4)	149 (32.0)	91 (30.8)	32 (28.5)	
High	452 (22.5)	509 (25.8)	1,190 (31.3)	481 (32.5)	150 (31.4)	25 (25.2)	76 (27.9)	143 (30.7)	73 (21.2)	33 (31.1)	
Education status											<0.001
≤6 years	1,016 (43.5)	578 (25.3)	879 (20.1)	288 (16.6)	109 (20.0)	51 (34.3)	38 (10.0)	56 (10.5)	39 (13.3)	18 (11.1)	
7–9 years	274 (13.3)	216 (11.1)	404 (10.8)	151 (10.4)	46 (10.1)	19 (21.7)	23 (6.0)	45 (10.5)	33 (11.9)	15 (15.1)	
10–12 years	481 (26.2)	577 (34.2)	1,314 (36.8)	517 (37.9)	175 (38.9)	32 (28.7)	116 (48.8)	185 (45.6)	152 (48.9)	55 (52.5)	
≥13 years	302 (17.0)	557 (29.3)	1,202 (32.3)	483 (35.1)	150 (31.0)	22 (15.4)	95 (35.3)	154 (33.4)	83 (25.9)	23 (21.3)	
Physical activity											<0.001
Yes	601 (31.1)	543 (28.4)	1,320 (35.5)	532 (38.6)	167 (37.1)	53 (39.6)	90 (32.5)	154 (34.3)	119 (39.0)	40 (38.6)	
No	1,472 (68.9)	1,385 (71.6)	2,479 (64.5)	907 (61.4)	313 (62.9)	71 (60.4)	182 (67.5)	286 (65.7)	188 (61.0)	71 (61.4)	
Smoking status											<0.001
Current smoker	31 (1.7)	60 (3.7)	89 (2.5)	46 (3.7)	24 (5.9)	12 (14.2)	30 (10.6)	61 (18.0)	71 (23.6)	40 (37.4)	
Ex-smoker	29 (1.6)	87 (5.0)	141 (4.1)	76 (5.4)	44 (10.1)	8 (10.9)	23 (8.9)	35 (7.9)	40 (14.0)	13 (10.6)	
Never smoker	2,013 (96.7)	1,781 (91.3)	3,569 (93.4)	1,317 (90.8)	412 (84.0)	104 (74.8)	219 (80.5)	344 (74.1)	196 (62.4)	58 (52.0)	
BMI											<0.001
<18.5 kg/m^2^	91 (4.3)	96 (5.8)	148 (4.0)	59 (4.2)	12 (2.1)	2 (1.5)	7 (3.5)	19 (3.2)	12 (3.5)	0 (0)	
18.5–24.9 kg/m^2^	1,291 (62.4)	1,266 (65.8)	2,611 (68.8)	1,018 (71.2)	358 (74.6)	85 (69.4)	180 (65.8)	281 (64.1)	196 (62.5)	75 (66.6)	
≥25 kg/m^2^	691 (33.3)	566 (28.5)	1,040 (27.2)	362 (24.6)	110 (23.2)	37 (29.2)	85 (30.7)	140 (32.7)	99 (34.0)	36 (33.4)	
Hypercholesterolemia											<0.001
Yes	476 (21.2)	326 (14.9)	575 (13.7)	190 (11.6)	59 (11.7)	31 (23.7)	36 (12.6)	57 (11.5)	32 (10.6)	18 (15.2)	
No	1,597 (78.8)	1,602 (85.1)	3,224 (86.3)	1,249 (88.5)	421 (88.3)	93 (76.4)	236 (87.4)	383 (88.5)	275 (89.4)	93 (84.8)	
Family history of DM											<0.001
Yes	202 (11.1)	292 (15.3)	700 (19.2)	266 (20.5)	91 (17.7)	18 (15.7)	57 (21.4)	76 (19.3)	62 (21.5)	18 (16.4)	
No	1,871 (88.9)	1,636 (84.7)	3,099 (80.8)	1,173 (79.5)	389 (82.3)	106 (84.3)	215 (78.6)	364 (80.7)	245 (78.5)	93 (83.6)	


DM, diabetes mellitus; IFG, impaired fasting glucose; BMI, body mass index.

^a^High-risk drinking was defined as alcohol consumption of ≥7 glasses on a single occasion for men and ≥5 glasses for women, and non-high risk drinking was defined as alcohol consumption of <7 glasses for men and <5 glasses for women.

^b^Continuous variables were calculated by using general linear models, and data are shown as mean (standard error). Categorical variables were calculated by using the chi-square test, and data are shown as unweighted number (weighted percentage).

Table 2 and Table 3 show the results of polychotomous logistic regressions performed to analyze the association of drinking patterns with IFG and DM in men and women. Model 1 (age adjusted) and model 2 (multivariable adjusted) showed similar patterns of significance in both men and women. In men, the multivariate adjusted ORs of IFG and DM were significantly higher in groups with high-risk drinking who drank on 2–4 days/month (IFG: OR 1.51; 95% CI, 1.13–2.04 and DM: OR 2.12; 95% CI, 1.20–3.77), groups with high-risk drinking who drank on 2–3 days/week (IFG: OR 1.79; 95% CI, 1.38–2.33 and DM: OR 1.78; 95% CI, 1.05–3.03), and groups with high-risk drinking who drank on ≥4 days/week (IFG: OR 2.24; 95% CI, 1.65–3.03 and DM: OR 2.98; 95% CI, 1.72–5.17) compared with groups without high-risk drinking who drank on ≤1 day/month. The average alcohol consumption (427.5 ± 5.3 g/month) of non-high-risk drinkers who drank 2–3 days/week was higher than that of high-risk drinkers who drank on 2–4 days/month (352.1 ± 5.0 g/month); however, their odds of IFG and DM were lower than those of high-risk drinkers who drank on 2–4 days/month. Non-high-risk drinkers who drank on ≥4 days/week also had higher odds of DM (OR 1.93; 95% CI, 1.09–3.42) (Table 2).

**Table 2.** Odds ratios of impaired fasting glucose and type 2 diabetes according to drinking patterns in men

Drinking frequency	Number of cases	Average alcohol consumption,^c^ g/month	Model 1^a^			

			IFG		DM	

		Mean (SE)	OR (95% CI)	*P* value	OR (95% CI)	*P* value
Lifetime abstainer	386	—	0.72 (0.50–1.02)	0.066	1.21 (0.66–2.20)	0.540
Past drinker	868	—	1.01 (0.79–1.29)	0.910	1.32 (0.82–2.12)	0.259
≤1/month						
Non-high-risk drinking	1,255	19.5 (0.5)	1		1	
High-risk drinking^d^	165	87.5 (3.7)	0.88 (0.56–1.38)	0.565	0.78 (0.31–1.97)	0.602
2–4/month						
Non-high-risk drinking	1,285	118.2 (1.5)	1.07 (0.86–1.33)	0.549	1.52 (0.97–2.39)	0.069
High-risk drinking	775	352.1 (5.0)	**1.38 (1.07–1.78)**	**0.012**	**1.98 (1.18–3.32)**	**0.010**
2–3/week						
Non-high-risk drinking	995	427.5 (5.3)	**1.33 (1.06–1.67)**	**0.015**	1.28 (0.78–2.09)	0.335
High-risk drinking	1,031	1,213.9 (14.7)	**1.74 (1.40–2.15)**	**<0.001**	**1.86 (1.16–3.00)**	**0.010**
≥4/week						
Non-high-risk drinking	552	803.8 (14.9)	1.28 (0.97–1.70)	0.082	**2.47 (1.48–4.10)**	**0.001**
High-risk drinking	563	2,492.4 (37.4)	**2.10 (1.63–2.72)**	**<0.001**	**3.10 (1.94–4.94)**	**<0.001**

Drinking frequency	Number of cases	Average alcohol consumption, g/month	Model 2^b^			

			IFG		DM	

		Mean (SE)	OR (95% CI)	*P* value	OR (95% CI)	*P* value

Lifetime abstainer	386	—	0.80 (0.55–1.15)	0.218	1.31 (0.71–2.42)	0.394
Past drinker	868	—	1.15 (0.89–1.50)	0.280	1.60 (0.98–2.61)	0.060
≤1/month						
Non-high-risk drinking	1,255	19.5 (0.5)	1		1	
High-risk drinking	165	87.5 (3.7)	0.96 (0.59–1.56)	0.865	0.83 (0.31–2.22)	0.717
2–4/month						
Non-high-risk drinking	1,285	118.2 (1.5)	0.94 (0.74–1.20)	0.613	1.24 (0.74–2.08)	0.413
High-risk drinking	775	352.1 (5.0)	**1.51 (1.13–2.04)**	**0.006**	**2.12 (1.20–3.77)**	**0.010**
2–3/week						
Non-high-risk drinking	995	427.5 (5.3)	1.10 (0.85–1.44)	0.470	0.87 (0.49–1.53)	0.620
High-risk drinking	1,031	1,213.9 (14.7)	**1.79 (1.38–2.33)**	**<0.001**	**1.78 (1.05–3.03)**	**0.033**
≥4/week						
Non-high-risk drinking	552	803.8 (14.9)	1.14 (0.84–1.56)	0.391	**1.93 (1.09–3.42)**	**0.024**
High-risk drinking	563	2,492.4 (37.4)	**2.24 (1.65–3.03)**	**<0.001**	**2.98 (1.72–5.17)**	**<0.001**

CI, confidence interval; DM, diabetes mellitus; IFG, impaired fasting glucose; OR, odds ratio; SE, standard error.

^a^Model 1: Adjusted for age.

^b^Model 2: Adjusted for age, body mass index, smoking status, household income, educational level, physical activity, family history of diabetes mellitus, hypercholesterolemia, and alcohol consumption amount in drinking days.

^c^Average alcohol consumption was calculated by using descriptive statistics.

^d^High-risk drinking was defined as alcohol consumption of ≥7 glasses on a single occasion for men and ≥5 glasses for women, and non-high risk drinking was defined as alcohol consumption of <7 glasses for men and <5 glasses for women.

**Table 3.** Odds ratios of impaired fasting glucose and type 2 diabetes according to drinking patterns in women

Drinking frequency	Number of cases	Average alcohol consumption,^c^ g/month	Model 1^a^			

			IFG		DM	

		Mean (SE)	OR (95% CI)	*P* value	OR (95% CI)	*P* value
Lifetime abstainer	2,073	—	1.04 (0.86–1.25)	0.686	1.06 (0.76–1.46)	0.745
Past drinker	1,928	—	0.93 (0.77–1.12)	0.442	1.03 (0.75–1.43)	0.844
≤1/month						
Non-high-risk drinking	3,799	12.5 (0.2)	1		1	
High-risk drinking^d^	272	57.4 (2.2)	1.02 (0.69–1.60)	0.939	0.57 (0.23–1.43)	0.233
2–4/month						
Non-high-risk drinking	1,439	73.6 (1.0)	**1.32 (1.09–1.60)**	**0.005**	0.86 (0.58–1.28)	0.468
High-risk drinking	440	228.0 (6.2)	**1.54 (1.10–2.16)**	**0.011**	1.14 (0.57–2.27)	0.707
2–3/week						
Non-high-risk drinking	480	262.5 (5.3)	1.32 (0.98–1.78)	0.072	1.18 (0.67–2.10)	0.564
High-risk drinking	307	831.7 (28.5)	**3.13 (2.25–4.36)**	**<0.001**	1.22 (0.49–3.05)	0.672
≥4/week						
Non-high-risk drinking	124	503.5 (21.8)	0.98 (0.57–1.69)	0.944	**0.18 (0.04–0.76)**	**0.020**
High-risk drinking	111	2,103.8 (105.2)	**2.14 (1.30–3.53)**	**0.003**	0.91 (0.28–2.98)	0.871

Drinking frequency	Number of cases	Average alcohol consumption g/month	Model 2^b^			

			IFG		DM	

		Mean (SE)	OR (95% CI)	*P* value	OR (95% CI)	*P* value

Lifetime abstainer	2,073	—	1.10 (0.91–1.32)	0.325	1.05 (0.76–1.45)	0.783
Past drinker	1,928	—	0.99 (0.82–1.20)	0.926	1.01 (0.72–1.42)	0.940
≤1/month						
Non-high-risk drinking	3,799	12.5 (0.2)	1		1	
High-risk drinking	272	57.4 (2.2)	1.00 (0.66–1.52)	0.988	0.43 (0.18–1.05)	0.063
2–4/month						
Non-high-risk drinking	1,439	73.6 (1.0)	**1.28 (1.05–1.56)**	**0.016**	0.97 (0.65–1.46)	0.891
High-risk drinking	440	228.0 (6.2)	**1.51 (1.04–2.21)**	**0.032**	0.81 (0.31–2.12)	0.669
2–3/week						
Non-high-risk drinking	480	262.5 (5.3)	1.26 (0.93–1.71)	0.133	1.49 (0.80–2.76)	0.205
High-risk drinking	307	831.7 (28.5)	**3.19 (2.20–4.64)**	**<0.001**	0.87 (0.38–1.98)	0.735
≥4/week						
Non-high-risk drinking	124	503.5 (21.8)	0.92 (0.55–1.55)	0.757	**0.18 (0.04–0.78)**	**0.022**
High-risk drinking	111	2,103.8 (105.2)	**2.23 (1.23–4.06)**	**0.009**	0.61 (0.15–2.54)	0.497

CI, confidence interval; DM, diabetes mellitus; IFG, impaired fasting glucose; OR, odds ratio; SE, standard error.

^a^Model 1: Adjusted for age.

^b^Model 2: Adjusted for age, body mass index, smoking status, household income, educational level, physical activity, family history of diabetes mellitus, hypercholesterolemia, and alcohol consumption amount in drinking days.

^c^Average alcohol consumption was calculated by using descriptive statistics.

^d^High-risk drinking was defined as alcohol consumption of ≥7 glasses on a single occasion for men and ≥5 glasses for women, and non-high risk drinking was defined as alcohol consumption of <7 glasses for men and <5 glasses for women.

In women, the multivariable adjusted ORs of IFG in the non-high-risk drinking group who drank on 2–4 days/month and high-risk drinking group who drank on 2–4 days/month (OR 1.51; 95% CI, 1.04–2.21), 2–3 days/week (OR 3.19; 95% CI, 2.20–4.64), and ≥4 days/week (OR 2.23; 95% CI, 1.23–4.06) were significantly higher compared with groups without high-risk drinking who drank on ≤1 day/month. Unlike men, female non-high-risk drinkers who drank on ≥4 days/week had lower ORs of DM (OR 0.18; 95% CI, 0.04–0.78) (Table 3).

Table 4 presents the ORs for IFG and DM according to average alcohol consumption from polychotomous logistic regression analysis. In men, the multivariate adjusted ORs of IFG and DM in groups who drank ≥5 g/day were significantly higher in a dose-response manner compared with groups who drank 0–4.9 g/day. In women, the multivariable adjusted ORs of IFG in those who drank ≥5 g/day were significantly higher; however, the ORs of DM were not significantly higher when compared with groups who drank 0–4.9 g/day. The significantly higher ORs for IFG and DM in men and IFG in women compared with the lowest alcohol consumption category in model 2 gradually attenuated compared with the ORs in model 1 (adjusted for age); however, the significance remained.

**Table 4.** Odds ratio for impaired fasting glucose and diabetes mellitus according to average alcohol consumption in polychotomous logistic regression analysis

	Men						Women					
	
Average alcohol consumption												

Model 1^a^	IFG			DM			IFG			DM		
			
	Number of cases	OR (95% CI)	*P* value	Number of cases	OR (95% CI)	*P* value	Number of cases	OR (95% CI)	*P* value	Number of cases	OR (95% CI)	*P* value
≥30 g/day	*n* = 462	**2.00 (1.66–2.40)**	**<0.001**	*n* = 95	**2.65 (1.88–3.74)**	**<0.001**	*n* = 42	**2.11 (1.42–3.15)**	**<0.001**	*n* = 4	1.33 (0.42–4.26)	0.627
15.0–29.9 g/day	*n* = 470	**1.50 (1.26–1.79)**	**<0.001**	*n* = 87	**1.89 (1.31–2.72)**	**0.001**	*n* = 90	**2.47 (1.82–3.35)**	**<0.001**	*n* = 9	0.83 (0.39–1.78)	0.632
5.0–14.9 g/day	*n* = 396	**1.29 (1.07–1.56)**	**0.008**	*n* = 83	**1.44 (1.02–2.05)**	**0.040**	*n* = 130	**1.37 (1.07–1.75)**	**0.013**	*n* = 21	1.01 (0.61–1.67)	0.978
0–4.9 g/day	*n* = 573	1		*n* = 109	1		*n* = 945	1		*n* = 227	1	
Past drinkers	*n* = 236	1.04 (0.83–1.30)	0.723	*n* = 55	1.23 (0.81–1.86)	0.332	*n* = 308	0.87 (0.73–1.03)	0.108	*n* = 87	1.07 (0.79–1.46)	0.650
Lifetime abstainers	*n* = 86	0.74 (0.53–1.03)	0.071	*n* = 21	1.12 (0.64–1.95)	0.693	*n* = 445	0.97 (0.82–1.16)	0.759	*n* = 126	1.10 (0.81–1.49)	0.537

Model 2^b^	IFG			DM			IFG			DM		

≥30 g/day	*n* = 462	**1.86 (1.54–2.26)**	**<0.001**	*n* = 95	**2.37 (1.67–3.36)**	**<0.001**	*n* = 42	**2.00 (1.32–3.02)**	**0.001**	*n* = 4	1.11 (0.35–3.48)	0.861
15.0–29.9 g/day	*n* = 470	**1.39 (1.17–1.66)**	**<0.001**	*n* = 87	**1.65 (1.14–2.40)**	**0.009**	*n* = 90	**2.36 (1.74–3.21)**	**<0.001**	*n* = 9	0.70 (0.32–1.56)	0.383
5.0–14.9 g/day	*n* = 396	**1.29 (1.06–1.56)**	**0.011**	*n* = 83	**1.44 (1.01–2.05)**	**0.043**	*n* = 130	**1.33 (1.03–1.71)**	**0.028**	*n* = 21	0.93 (0.55–1.56)	0.786
0–4.9 g/day	*n* = 573	1		*n* = 109	1		*n* = 945	1		*n* = 227	1	
Past drinkers	*n* = 236	1.07 (0.85–1.34)	0.561	*n* = 55	1.24 (0.82–1.87)	0.312	*n* = 308	0.89 (0.75–1.06)	0.175	*n* = 87	1.08 (0.79–1.49)	0.617
Lifetime abstainers	*n* = 86	0.74 (0.53–1.04)	0.078	*n* = 21	1.03 (0.58–1.82)	0.933	*n* = 445	0.99 (0.84–1.17)	0.900	*n* = 126	1.12 (0.83–1.52)	0.466

CI, confidence interval; DM, diabetes mellitus; IFG, impaired fasting glucose; OR, odds ratio; SE, standard error.

^a^Model 1: Adjusted for age.

^b^Model 2: Adjusted for age, body mass index, smoking status, household income, educational level, physical activity, family history of diabetes mellitus, and hypercholesterolemia.

## DISCUSSION

Our study showed that higher average alcohol consumption is associated with higher odds of IFG and DM in men and IFG in women compared with men and women in the lowest alcohol consumption category. Moreover, when compared with occasional non-high-risk drinkers, the ORs for IFG were higher among male and female occasional high-risk drinkers, and the ORs for DM were higher among male occasional high-risk drinkers. The patterns of ORs for DM among nearly daily non-high-risk drinkers differed by sex: men had higher ORs, but women had lower ORs.

Outcomes of the analyses of the association between average alcohol consumption and DM are in accordance with reports of other studies. Heavy alcohol consumption (>50 mL/day of ethanol) was associated with an increased risk of type 2 DM in lean men (BMI ≤22 kg/m^2^) in a cohort of 6,362 Japanese men aged 35–61 years who did not have diabetes.^17^ The relative risk of type 2 DM is increased in men who drink >60 g/day and in women who drink >50 g/day compared with lifetime abstainers in one meta-analysis.^18^

Furthermore, the association between drinking patterns and DM in this study was similar to that of previous studies. Hodge et al^19^ showed that the risk of DM increased in men who drank ≥210 g alcohol during 1–3 days compared with men who consumed no alcohol in the week before baseline, whereas the risk of DM did not increase in men who drank the same amount of alcohol during more days in a prospective study of 36,527 adults aged 40–69 at baseline. In a study on 1,650 Japanese men without diabetes, binge drinking (≥3 drinks per occasion) significantly increased the risk of DM regardless of frequency, compared with those who drank <1 drink per occasion.^8^ In a prospective cohort of 12,261 middle-aged participants of the Atherosclerosis Risk in Communities Study (1990–1998), the risk of diabetes increased significantly only in men who drank >21 drinks per week compared with men who drank ≤1 drink per week^20^; however, a significant association was not found in women. In another prospective study among 109,690 American women aged 25–42 years, light-to-moderate drinking was associated with a lower risk of DM, and the risk was not increased even in the highest category of alcohol consumption.^21^

In both men and women, non-high-risk drinkers who drank 2–3 days/week did not have significantly higher odds of IFG and DM, unlike high-risk drinkers who drank 2–4 days/month, although the first group had a higher amount of average alcohol consumption. The association between high-risk drinking and IFG and DM can be explained via several mechanisms. Acute liver damage caused by alcohol could induce defects in glucose homeostasis, which could cause impairment in glucose control.^22^ In addition, excessive alcohol consumption in the short term can stimulate appetite and lead to the development of obesity by inhibiting the increase of postprandial blood glucose, which, in turn, inhibits gluconeogenesis.^23^ Excessive alcohol consumption in the long term might also contribute to an increase in fasting blood glucose by increasing the serum level of 2,3-butanediol and 1,2-propanediol, both of which reduce the use of plasma glucose in skeletal muscle and adipocytes by up to 30% and increase insulin resistance.^24^ Furthermore, alcohol intoxication increases the activity of sympathetic nerves,^25^ thereby increasing insulin resistance.^26^ Moreover, binge drinking could induce insulin resistance by impairing insulin action in the hypothalamus.^27^ The association of the frequency of high-risk drinking with IFG and DM observed in this study could be explained by these mechanisms.

Our study also showed the different direction of associations between nearly daily non-high-risk drinking and DM among men and women; men had higher ORs of DM, but women had lower ORs. A previous meta-analysis also showed that the reduction in the risk of DM among moderate drinkers may be confined to women.^28^ Also, the association between high-risk drinking and DM was different among men and women in our study; men had higher ORs of DM, but women did not. There are several possible explanations for these findings. First and foremost, the statistical power was not adequate to investigate the association between DM and drinking pattern in women. The proportion of DM subjects with high-risk drinking pattern was much lower for women than men, thereby reducing the power to detect an association. Second, in a prospective cohort of 12,261 middle-aged participants of the Atherosclerosis Risk in Communities Study (1990–1998), men tended to drink spirits whereas women were more likely to consume wine.^20^ Persons who prefer wine tend to have healthier behaviors than those who prefer spirits.^29^ This sex-based difference in alcoholic beverage preference might also have affected our results. Furthermore, in the study that reported the results from the Medical Research Council National Survey of Health and Development (the 1946 British Cohort), women who felt guilty about their alcohol consumption were more likely to underestimate their drinking compared with women who did not feel guilty about their drinking behavior; however, this effect was not apparent in men.^30^ In Korea particularly, alcohol consumption is considered essential for business, social gatherings, and promoting enduring friendships. There is public tolerance for drunkenness and mistakes committed under the influence of alcohol; however, this social acceptance is limited only to men.^31^ This gender-related prejudice and feelings of guilt about alcohol consumption could make women underreport their drinking behavior. Lastly, endogenous sex hormones might affect the glycemic status in men and women differently. In one meta-analysis, men who had higher testosterone levels had a lower risk of type 2 diabetes, whereas testosterone increased the risk of diabetes in women.^32^ In another study among 46 male alcohol abusers aged 20–40 years, alcohol abusers had significantly low plasma testosterone levels.^33^ These differences could have affected the results of our study.

This study has several limitations. First, the low number of female DM participants with high-risk drinking limited the statistical power. Second, we could not use the results of the oral glucose tolerance test to diagnose IFG or DM, as these are not available in the KNHANES study. Therefore, many participants with undiagnosed diabetes may have been missed. Additionally, since this was a cross-sectional study, we were unable to verify causal relationships of drinking patterns with IFG and DM. Furthermore, because the history of alcohol drinking was self-reported, recall bias may be present. Also, we could not classify the type of DM because this information was missing in the KNHANES study. However, we could assume that most of the patients with diabetes in our current study had type 2 DM, as the prevalence of type 1 DM in Korea is very low^34^ and our study subjects were older than 30 years. In addition, as HbA1c was tested only in subjects in whom DM was diagnosed in 2010, some patients with undiagnosed diabetes may have been missed. Last but not least, since the administration of the survey about the amount of alcohol consumed in relation to different alcoholic beverages began from 2012, we could not analyze the association of the frequency of alcohol consumption with IFG or DM in relation to different types of alcoholic beverages.

Regardless of these limitations, this study has strengths. First, we considered drinking patterns in the investigation of the association of alcohol consumption with IFG or DM. Considering the high prevalence of alcohol abuse among Korean adults,^35^ drinking patterns, as well as average alcohol consumption, are important factors when investigating the adverse effects of alcohol consumption. Second, we included IFG as an outcome variable in addition to DM. This was necessary because several studies have estimated that up to 70% of persons with IFG will eventually develop diabetes,^12^^,^^36^ so patients with IFG who have high-risk drinking patterns should receive education to enable them modify their alcohol consumption behavior and reduce their risk of developing diabetes. In addition, to minimize the sick quitter effect, we excluded from our analysis those subjects who already had a diagnosis of diabetes. Lastly, all analyses in our study were weighted to represent the Korean population, and various confounding factors, such as age and lifestyles, were adjusted. Thus, our study findings can be generalized to include the entire adult population of Korea.

In conclusion, higher average alcohol consumption is associated with higher odds of IFG and DM in men and IFG in women. Moreover, when compared with occasional non-high-risk drinkers, the ORs for IFG were higher among male and female occasional high-risk drinkers, and the ORs for DM were higher among male occasional high-risk drinkers. Therefore, alcohol control policies should focus both on average alcohol consumption and high-risk drinking patterns to reduce the development of DM.

## References

[1] Almdal T, Scharling H, Jensen JS, Vestergaard H The independent effect of type 2 diabetes mellitus on ischemic heart disease, stroke, and death: a population-based study of 13,000 men and women with 20 years of follow-up. Arch Intern Med. 2004;164:1422–1426. 10.1001/archinte.164.13.1422 15249351

[2] Kannel WB, McGee DL Diabetes and cardiovascular disease. The Framingham study. JAMA. 1979;241:2035–2038. 10.1001/jama.1979.03290450033020 430798

[3] Gu K, Cowie CC, Harris MI Diabetes and decline in heart disease mortality in US adults. JAMA. 1999;281:1291–1297. 10.1001/jama.281.14.1291 10208144

[4] Young BA, Lin E, Von Korff M, Diabetes complications severity index and risk of mortality, hospitalization, and healthcare utilization. Am J Manag Care. 2008;14:15–23. 18197741 PMC3810070

[5] Knowler WC, Barrett-Connor E, Fowler SE, ; Diabetes Prevention Program Research Group Reduction in the incidence of type 2 diabetes with lifestyle intervention or metformin. N Engl J Med. 2002;346(6):393–403. 10.1056/NEJMoa012512 11832527 PMC1370926

[6] Carlsson S, Hammar N, Grill V, Kaprio J Alcohol consumption and the incidence of type 2 diabetes: a 20-year follow-up of the Finnish twin cohort study. Diabetes Care. 2003;26:2785–2790. 10.2337/diacare.26.10.2785 14514580

[7] Wei M, Gibbons LW, Mitchell TL, Kampert JB, Blair SN Alcohol intake and incidence of type 2 diabetes in men. Diabetes Care. 2000;23:18–22. 10.2337/diacare.23.1.18 10857962

[8] Heianza Y, Arase Y, Saito K, Role of alcohol drinking pattern in type 2 diabetes in Japanese men: the Toranomon Hospital Health Management Center Study 11 (TOPICS 11). Am J Clin Nutr. 2013;97:561–568. 10.3945/ajcn.112.043364 23343972

[9] Sato KK, Hayashi T, Harita N, Relationship between drinking patterns and the risk of type 2 diabetes: the Kansai Healthcare Study. J Epidemiol Community Health. 2012;66:507–511. 10.1136/jech.2010.109777 21131305

[10] Conigrave KM, Hu BF, Camargo CA Jr, Stampfer MJ, Willett WC, Rimm EB A prospective study of drinking patterns in relation to risk of type 2 diabetes among men. Diabetes. 2001;50:2390–2395. 10.2337/diabetes.50.10.2390 11574424

[11] Hong SW, Linton JA, Shim JY, Kang HT High-risk drinking is associated with a higher risk of diabetes mellitus in Korean men, based on the 2010–2012 KNHANES. Alcohol. 2015;49:275–281. 10.1016/j.alcohol.2015.02.004 25920001

[12] Vendrame F, Gottlieb PA Prediabetes: prediction and prevention trials. Endocrinol Metab Clin North Am. 2004;33:75–92, ix. 10.1016/j.ecl.2003.12.006 15053896

[13] Kweon S, Kim Y, Jang MJ, Data resource profile: the Korea National Health and Nutrition Examination Survey (KNHANES). Int J Epidemiol. 2014;43:69–77. 10.1093/ije/dyt228 24585853 PMC3937975

[14] Obesity: preventing and managing the global epidemic. Report of a WHO consultation. World Health Organ Tech Rep Ser. 2000;894:i–xii, 1–253. 11234459

[15] Roerecke M, Rehm J The cardioprotective association of average alcohol consumption and ischaemic heart disease: a systematic review and meta-analysis. Addiction. 2012;107:1246–1260. 10.1111/j.1360-0443.2012.03780.x 22229788 PMC3348338

[16] World Health Organization. *International Guide for Monitoring Alcohol Consumption and Related Harm*. 2000.

[17] Tsumura K, Hayashi T, Suematsu C, Endo G, Fujii S, Okada K Daily alcohol consumption and the risk of type 2 diabetes in Japanese men: the Osaka Health Survey. Diabetes Care. 1999;22:1432–1437. 10.2337/diacare.22.9.1432 10480505

[18] Baliunas DO, Taylor BJ, Irving H, Alcohol as a risk factor for type 2 diabetes: a systematic review and meta-analysis. Diabetes Care. 2009;32:2123–2132. 10.2337/dc09-0227 19875607 PMC2768203

[19] Hodge AM, English DR, O’Dea K, Giles GG Alcohol intake, consumption pattern and beverage type, and the risk of Type 2 diabetes. Diabet Med. 2006;23:690–697. 10.1111/j.1464-5491.2006.01864.x 16759314

[20] Kao WH, Puddey IB, Boland LL, Watson RL, Brancati FL Alcohol consumption and the risk of type 2 diabetes mellitus: atherosclerosis risk in communities study. Am J Epidemiol. 2001;154:748–757. 10.1093/aje/154.8.748 11590088

[21] Wannamethee SG, Camargo CA Jr, Manson JE, Willett WC, Rimm EB Alcohol drinking patterns and risk of type 2 diabetes mellitus among younger women. Arch Intern Med. 2003;163:1329–1336. 10.1001/archinte.163.11.1329 12796069

[22] Howard AA, Arnsten JH, Gourevitch MN Effect of alcohol consumption on diabetes mellitus: a systematic review. Ann Intern Med. 2004;140:211–219. 10.7326/0003-4819-140-6-200403160-00011 14757619

[23] Yokoyama H, Hiroshi H, Ohgo H, Hibi T, Saito I Effects of excessive ethanol consumption on the diagnosis of the metabolic syndrome using its clinical diagnostic criteria. Intern Med. 2007;46:1345–1352. 10.2169/internalmedicine.46.6196 17827831

[24] Xu D, Dhillon AS, Abelmann A, Croft K, Peters TJ, Palmer TN Alcohol-related diols cause acute insulin resistance in vivo. Metabolism. 1998;47:1180–1186. 10.1016/S0026-0495(98)90320-1 9781618

[25] van de Borne P, Mark AL, Montano N, Mion D, Somers VK Effects of alcohol on sympathetic activity, hemodynamics, and chemoreflex sensitivity. Hypertension. 1997;29:1278–1283. 10.1161/01.HYP.29.6.1278 9180629

[26] Mancia G, Bousquet P, Elghozi JL, The sympathetic nervous system and the metabolic syndrome. J Hypertens. 2007;25:909–920. 10.1097/HJH.0b013e328048d004 17414649

[27] Lindtner C, Scherer T, Zielinski E, Binge drinking induces whole-body insulin resistance by impairing hypothalamic insulin action. Sci Transl Med. 2013;5:170ra14. 10.1126/scitranslmed.3005123 23363978 PMC3740748

[28] Knott C, Bell S, Britton A Alcohol consumption and the risk of type 2 diabetes: a systematic review and dose-response meta-analysis of more than 1.9 million individuals from 38 observational studies. Diabetes Care. 2015;38:1804–1812. 10.2337/dc15-0710 26294775

[29] Klatsky AL, Friedman GD, Armstrong MA The relationships between alcoholic beverage use and other traits to blood pressure: a new Kaiser Permanente study. Circulation. 1986;73:628–636. 10.1161/01.CIR.73.4.628 3948365

[30] Ely M, Hardy R, Longford NT, Wadsworth ME. *Methods of estimating individual levels of alcohol consumption in the general population*. UK Alcohol Education and Research Council (AERC) Final Report; 2001.

[31] Kim W, Kim S Women’s alcohol use and alcoholism in Korea. Subst Use Misuse. 2008;43:1078–1087. 10.1080/10826080801914212 18649231

[32] Ding EL, Song Y, Malik VS, Liu S Sex differences of endogenous sex hormones and risk of type 2 diabetes: A systematic review and meta-analysis. JAMA. 2006;295:1288–1299. 10.1001/jama.295.11.1288 16537739

[33] Maneesh M, Dutta S, Chakrabarti A, Vasudevan DM Alcohol abuse-duration dependent decrease in plasma testosterone and antioxidants in males. Indian J Physiol Pharmacol. 2006;50:291–296. 17193902

[34] Karvonen M, Viik-Kajander M, Moltchanova E, Libman I, LaPorte R, Tuomilehto J Incidence of childhood type 1 diabetes worldwide. Diabetes Mondiale (DiaMond) Project Group. Diabetes Care. 2000;23:1516–1526. 10.2337/diacare.23.10.1516 11023146

[35] World Health Organization. *Global status report on alcohol and health*. Geneva; 2014.

[36] Shaw JE, Zimmet PZ, de Courten M, Impaired fasting glucose or impaired glucose tolerance. What best predicts future diabetes in Mauritius? Diabetes Care. 1999;22:399–402. 10.2337/diacare.22.3.399 10097917

